# One size doesn’t fit all: methodological reflections in conducting community-based behavioural science research to tailor COVID-19 vaccination initiatives for public health priority populations

**DOI:** 10.1186/s12889-024-18270-x

**Published:** 2024-03-13

**Authors:** Guillaume Fontaine, Maureen Smith, Tori Langmuir, Karim Mekki, Hanan Ghazal, Elizabeth Estey Noad, Judy Buchan, Vinita Dubey, Andrea M. Patey, Nicola McCleary, Emily Gibson, Mackenzie Wilson, Amjad Alghamyan, Kateryna Zmytrovych, Kimberly Thompson, Jacob Crawshaw, Jeremy M. Grimshaw, Trevor Arnason, Jamie Brehaut, Susan Michie, Melissa Brouwers, Justin Presseau

**Affiliations:** 1 Centre for Implementation Research, Clinical Epidemiology Program, Ottawa Hospital Research Institute, 501 Smyth Rd, Ottawa, ON K1H 8L6 Canada; 2 Department of Medicine, University of Ottawa, 451 Smyth Rd, Ottawa, ON K1H 8M5 Canada; 3 Citizen Partner, Ottawa, ON Canada; 4 Ottawa Public Health, 100 Constellation Dr, Nepean, ON K2G 6J8 Canada; 5 Peel Public Health, 7120 Hurontario St, Mississauga, ON L5W 1N4 Canada; 6 Toronto Public Health, City Hall, 100 Queen St W, Toronto, ON M5H 2N2 Canada; 7 School of Epidemiology and Public Health, 600 Peter Morand Crescent, Ottawa, ON K1G 5Z3 Canada; 8 School of Rehabilitation Therapy, Queen’s University, Louise D Acton Building, 31 George St, Kingston, ON K7L 3N6 Canada; 9 University of Ottawa, Ottawa, ON Canada; 10 Citizen Partner, Mississauga, ON Canada; 11 The Hospital for Sick Children (SickKids), Toronto, ON Canada; 12 McMaster University, 1280 Main St W, Hamilton, ON L8S 4L8 Canada; 13 Centre for Behaviour Change, University College London, Gower St, London, WC1E 6BT UK; 14 School of Psychology, University of Ottawa, 136 Jean-Jacques Lussier Vanier Hall, Ottawa, ON K1N 6N5 Canada

**Keywords:** Community-based participatory research, Community engagement, Citizen engagement, COVID-19, Behavioural science, Vaccination

## Abstract

**Background:**

Promoting the uptake of vaccination for infectious diseases such as COVID-19 remains a global challenge, necessitating collaborative efforts between public health units (PHUs) and communities. Applied behavioural science can play a crucial role in supporting PHUs’ response by providing insights into human behaviour and informing tailored strategies to enhance vaccination uptake. Community engagement can help broaden the reach of behavioural science research by involving a more diverse range of populations and ensuring that strategies better represent the needs of specific communities. We developed and applied an approach to conducting community-based behavioural science research with ethnically and socioeconomically diverse populations to guide PHUs in tailoring their strategies to promote COVID-19 vaccination. This paper presents the community engagement methodology and the lessons learned in applying the methodology.

**Methods:**

The community engagement methodology was developed based on integrated knowledge translation (iKT) and community-based participatory research (CBPR) principles. The study involved collaboration with PHUs and local communities in Ontario, Canada to identify priority groups for COVID-19 vaccination, understand factors influencing vaccine uptake and co-design strategies tailored to each community to promote vaccination. Community engagement was conducted across three large urban regions with individuals from Eastern European communities, African, Black, and Caribbean communities and low socioeconomic neighbourhoods.

**Results:**

We developed and applied a seven-step methodology for conducting community-based behavioural science research: (1) aligning goals with system-level partners; (2) engaging with PHUs to understand priorities; (3) understanding community strengths and dynamics; (4) building relationships with each community; (5) establishing partnerships (community advisory groups); (6) involving community members in the research process; and (7) feeding back and interpreting research findings. Research partnerships were successfully established with members of prioritized communities, enabling recruitment of participants for theory-informed behavioural science interviews, interpretation of findings, and co-design of targeted recommendations for each PHU to improve COVID-19 vaccination uptake. Lessons learned include the importance of cultural sensitivity and awareness of sociopolitical context in tailoring community engagement, being agile to address the diverse and evolving priorities of PHUs, and building trust to achieve effective community engagement.

**Conclusion:**

Effective community engagement in behavioural science research can lead to more inclusive and representative research. The community engagement approach developed and applied in this study acknowledges the diversity of communities, recognizes the central role of PHUs, and can help in addressing complex public health challenges.

**Supplementary Information:**

The online version contains supplementary material available at 10.1186/s12889-024-18270-x.

## Background

With over 769 million confirmed cases and 6.9 million deaths, the coronavirus disease 2019 (COVID-19) continues to pose significant public health challenges across the globe [1]. Health systems, economies, and social structures have been widely impacted by COVID-19, leading to changes in public health policy in many countries [2–4]. Epidemiological data shows that the infection can affect people of all ages, although older adults and those with underlying medical conditions are at increased risk of severe illness and death [5]. Certain racial and ethnic groups, as well as those living in densely populated areas, are also at increased risk [6–9]. For example, individuals of Black and Asian ethnicity are at increased risk of COVID-19 infection compared to white individuals [10]. Several factors contribute to higher rates of infection in ethnic minority groups, including racism and structural discrimination, lower socioeconomic status, living in larger and multi-generational households with shared facilities, and being employed in essential jobs with frequent exposure to infection and proximity to others [10].

Addressing these inequities requires multifaceted interventions, from mitigating structural disparities to promoting the uptake of public health and social measures aimed at preventing the transmission and spread of COVID-19 [10, 11]. While a wide range of measures have been recommended by the World Health Organization [12], the challenge lies in their effective implementation, especially concerning vaccination. Globally, promoting COVID-19 vaccination uptake (and boosters) during the ongoing (and constantly evolving) pandemic remains a challenge [13–15]. This is complicated by the politicization of COVID-19, the pervasive spread of misinformation within social media platforms, the mistrust of scientific authorities and institutions, cultural norms related to family, peers, religious leaders, and community leaders, and vaccine confidence, which have influenced behaviours towards these health measures and vaccination efforts, hindering their effectiveness [16–19]. Health inequities for structurally marginalized Canadians were exacerbated by COVID-19, underscoring the need for community-specific and responsive solutions [20–22]. These solutions should be based on an understanding of the unique barriers and facilitators to information dissemination and uptake within different communities to ensure their relevance and acceptability [20, 23, 24]. Factors such as language barriers, socioeconomic status, access to technology, and historical experiences with healthcare systems can significantly impact the relevance and acceptability of public health measures [20, 23, 24]. Therefore, addressing the health inequities exacerbated by the pandemic requires a comprehensive approach that prioritizes responsive solutions, ensuring that these are tailored to the assets, needs and challenges of each community.

In Ontario, Canada, Public Health Units (PHUs) have been at the forefront of the pandemic, battling not only the disease but also facing operational, ethical and communication challenges [25]. Ontario PHUs are the agencies responsible for delivery of local public health programs and services. One of the main challenges has been the scale of the pandemic, highlighting critical weaknesses in health systems. These weaknesses include limitations in the capacity and flexibility of service delivery with PHUs struggling to manage the large number of cases; inconsistencies in decision-making and coordination at different levels of the health system and government; and the significant strain on the health workforce revealing shortages and the need for additional training in epidemic management [26]. PHUs navigated complex ethical and political issues related to the pandemic response, such as balancing individual rights and public health priorities, as well as tensions between government and PHUs [27, 28]. In Canada, while the federal government provides overall guidance, PHUs, operating at the municipal or regional level, are primarily funded and governed by provincial or territorial governments [29]. This can lead to discrepancies in resource allocation, priorities, and decision-making processes, impacting public health initiatives. PHUs have also faced challenges in communicating with the public, especially dispelling misinformation and countering increasing mistrust [30]. There is a need for increased collaboration and coordination among public health authorities at the global, national and sub-national levels to share best practices, resources and expertise; PHUs need access to timely and reliable data to inform their decision-making and response efforts.

Applied behavioural science has the potential to play an important role in supporting PHUs’ programs and strategies during public health emergencies such as the COVID-19 pandemic [31, 32]. Behavioural science provides understanding of how key healthcare actors—including individuals, communities, healthcare professionals, and policy-makers—interact within health-related areas. It enables better knowledge about the attitudes and beliefs, and decision-making processes of these actors, and understanding of the underlying processes and mechanisms that drive specific health-related behaviours [33, 34]. Behavioural science also provides evidence about effective (and just as importantly – ineffective) ways to support behaviour change [35, 36]. Evidence-based solutions grounded in social and behavioural science have been shown to be more effective and sustainable in the long run [32, 37]. This is key for PHUs to design and implement more effective interventions to prevent the spread of COVID-19 [33, 34]. An area where behavioural science has provided important evidence is the factors that influence vaccination uptake [38]. By drawing on this evidence, PHUs could develop more effective campaigns and interventions that resonate with the public and encourage vaccination uptake [31]. PHUs have long-standing partnerships with specific communities, which could be leveraged to introduce behavioural science to develop effective solutions.

Community engagement has been integral to the pandemic response [39]. A recent review revealed a disconnect between communities and government in disseminating public health information during the pandemic [40]. To bridge this gap, engaging with local communities and community organizations to address specific health information needs and provide tailored strategies to various groups and communities can help in re-establishing public trust, enhancing inclusivity and increasing uptake of health information from credible sources [40]. More specifically, community engagement can support counter-messaging to address circulating misinformation, designing impactful messaging appeals, and identifying trusted health messengers within the community [22, 41–43]. Achieving a balance between public health priorities, which require urgent action, and building trust with communities, which requires time and resources, is challenging but necessary for effective communication and increased uptake of health information from credible sources [44]. There is a need to conduct community-based, behavioural science research with marginalized populations to identify community-specific and responsive solutions.

There is a knowledge gap in understanding how effective collaborations and relationships can be established between PHUs and communities to conduct behavioural science research. This paper presents the methodology we developed for community engagement with ethnically and socioeconomically diverse populations in Ontario, Canada to conduct behavioural science research to guide PHUs in their strategies to promote COVID-19 vaccination uptake. We share lessons learnt in applying the methodology, covering key considerations for fostering effective research partnerships with communities.

## Methods

### Study design and context

This is a nested study within the OPTimise project, a responsive and agile platform enabling PHUs to apply behavioural science-informed strategies in ways that reflect their evolving priorities and complement their existing efforts (hereafter ‘OPTimise Platform’). Partnerships were established with PHUs, community leaders and residents in two large cities (Ottawa, Toronto) and one region including two cities and a town (the region of Peel, including Mississauga, Brampton and Caledon) in Ontario, Canada. The OPTimise Platform was developed to generate generate an understanding of what influences individuals to engage in specific behaviours (e.g., getting vaccinated). This was used to create strategies to promote uptake of these behaviours in ways that reflect the realities of priority groups in each setting, including historically excluded and equity-denied groups. The approach, while comprehensive, was designed to be adaptable to time-sensitive situations, notably via the use of rapid analysis approach for the interviews conducted with residents. The OPTimise Platform was approved by the Ottawa Health Sciences Network Research Ethics Board (#20,200,285-01H). The research team included behavioural scientists, health services researchers, and a citizen partner with extensive experience in COVID-19 citizen engagement. Table 1 presents the terms used to designate the different community-based individuals involved in the OPTimise Platform. In this paper, we focus on the methodology we developed for community engagement, and the lessons learnt in applying the methodology. We discuss decisions that we made to collaborate effectively with PHUs, tackle the broader context of the politicization of COVID-19 and the polarization of views regarding the issue, how we built trust and ensured that the voices of community members were captured.

**Table 1:** Terms used to designate community-based individuals in the OPTimise platform

Term	Definition
***Citizen partner (MS)***	Patient with extensive experience in COVID-19 citizen engagement recruited during the development of the grant application to provide high-level input and support throughout the project, including reviewing all project materials and co-leading community engagement.
***Community member***	Umbrella term designating all individuals from a community engaged in the project, including community leaders and residents.
***Community leader***	Individuals from the communities of interest in the OPTimise Platform holding leadership or professional roles in various organizations serving their community (e.g., cultural community organization, community health centre).
***Community resident***	Individuals from the communities of interest in the OPTimise Platform.

### Guiding principles

The overall project was based on the principles of integrated knowledge translation (iKT) [45, 46] and the approach to community engagement drawn from the field of community-based participatory research (CBPR) [22, 43, 47]. iKT is a model of research involving collaboration between researchers and knowledge users (KUs; such as policymakers, healthcare providers, or community members) throughout the research process [45, 46]. This approach emphasizes the integration of knowledge generation and application, ensuring that research is directly relevant and useful [45, 46]. CBPR is a research approach that involves community members as active partners in the research process [22, 43, 45]. It seeks to address community-identified issues and work towards solutions collaboratively with the community [48].

iKT and CBPR converge towards a shared objective: the collaborative generation of knowledge that arises from the expertise of KUs and researchers and that is directly applicable and beneficial to the community and KUs involved [45, 49]. Previous research has shown that iKT and CBPR successfully support production of culturally and logistically appropriate research, recruitment of participants to projects and interventions, and capacity building of academic and community partners [46, 50]. These approaches can also enable conflict resolution and negotiation processes, sustain project goals beyond funded time frames, and generate systemic changes [46, 50]. Immunization research has shown participatory community engagement to be cost-effective, increase vaccine uptake and reduce healthcare resources needed to achieve high vaccination coverage in different contexts [51–53].

### Community engagement process

**Fig. 1:**
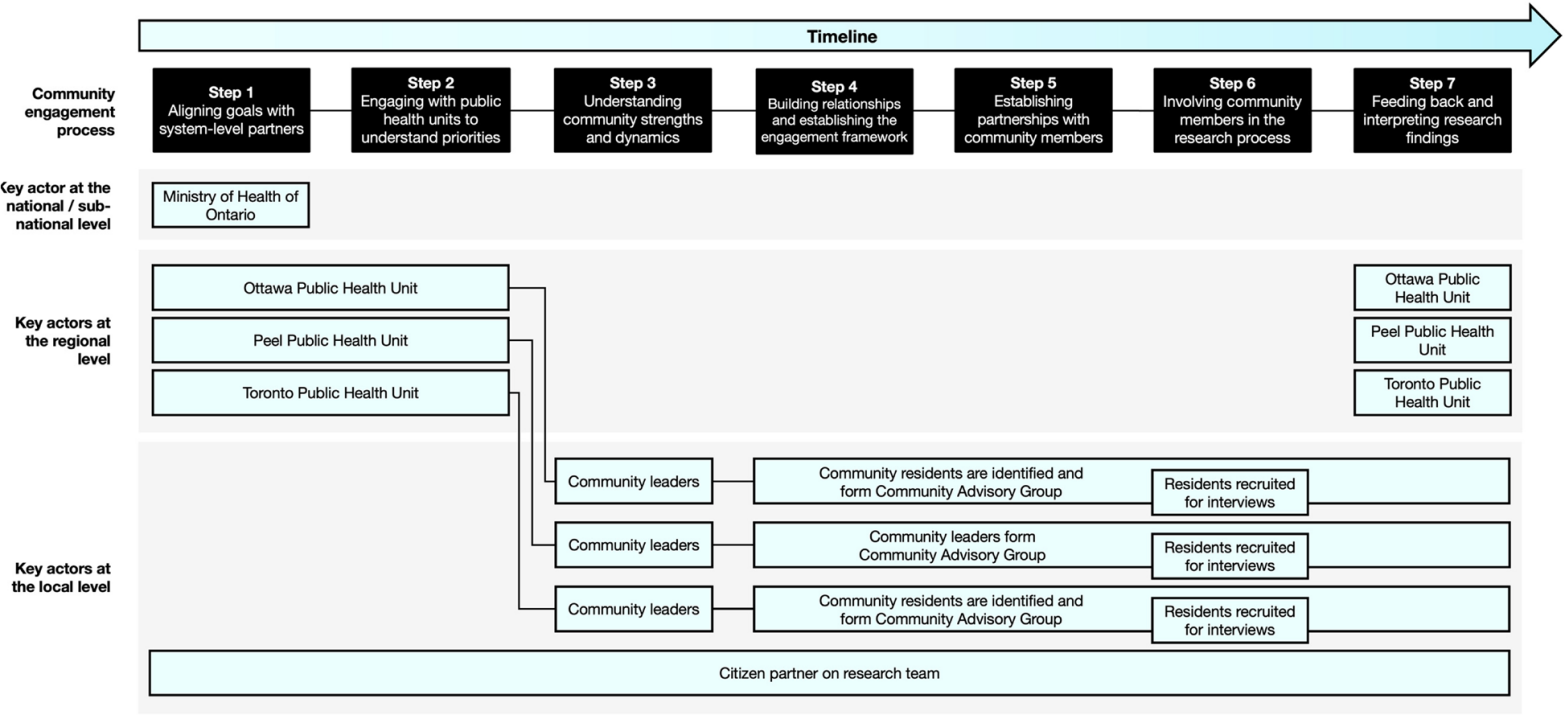
Illustration of the community engagement process alongside key actors

As presented in Fig. 1, we developed and applied a seven-step approach to community engagement: (1) aligning goals with system-level partners; (2) engaging with PHUs to understand priorities; (3) understanding community strengths and dynamics; (4) building relationships with each community and establishing the community engagement framework; (5) establishing partnerships with community members; (6) involving community members in the research process; and (7) feeding back and interpreting the research findings.

#### Step 1 – Aligning goals with system-level partners

To initiate community engagement, the research team began by meeting with decision-makers of system-level partners—Ministry of Health of Ontario and Ottawa, Peel and Toronto PHUs—to assess needs and establish shared goals. Discussions led the Ottawa, Peel and Toronto PHUs to identify a need for a behavioural science approach to inform PHU activities to promote vaccination at a community-level. This step occurred during the second year of the COVID-19 pandemic, when the first few waves had passed, and vaccines were readily available. On a societal level, some of the trust that characterized the early stages of the pandemic was eroding or lost by this point [54, 55]. For example, some people felt forced to take the vaccine or were hesitant to do so, and others were apprehensive about how recommendations regarding vaccination were constantly evolving [38, 56, 57].

#### Step 2 – Engaging with PHUs to understand priorities

During this step, the research team had to develop an understanding of the goals and needs of PHUs. The research team engaged with PHUs to understand how regional priorities varied due to differences in population density, demographics, health status, political and economic structures, healthcare systems and resource access. Additionally, the team aimed to identify which doses in a vaccination series were of greatest interest to the PHUs, considering that individuals’ psychological and behavioural states are likely to differ at each stage, influencing the assessment of barriers and facilitators to vaccination in the next phase of the study [36, 38]. Working with PHUs, we employed a prioritization matrix to determine the focus on specific vaccination doses and communities (see Supplementary Material 1).

Two PHUs (Peel and Toronto) identified getting the first dose of the COVID-19 vaccine as their initial priority. The Peel PHU initially prioritised individuals between the ages 30 and 49 years who are members of Eastern European communities (e.g., Polish, Ukrainian, or Russian), and later broadened it to individuals from Eastern European heritage, ages 18 years or older and from any neighbourhood. The Toronto PHU prioritised individuals ages 18 years or older who are members of African, Black, and Caribbean communities from five neighbourhoods with the lowest rates of vaccination. Peel and Toronto also prioritised the third or “booster” dose later in the year. The Ottawa PHU prioritised the 3rd dose and individuals ages 18 years or older from five fifth-quintile socioeconomic status neighbourhoods (i.e., low-income).

#### Step 3 – Understanding community strengths and dynamics

To understand the strengths (sociocultural, health-seeking) and vaccination behavioural dynamics of communities prioritized by PHUs, the research team first held meetings and engaged with community leaders in Ottawa, Peel Region and Toronto. This was crucial because each neighbourhood has a distinct community and public health infrastructure, leading to variations in sociocultural practices and healthcare behaviours. Furthermore, perceptions and attitudes towards vaccination varied markedly across communities, potentially influencing the engagement process. In parallel, we conducted a detailed environmental scan to identify how the three PHUs promoted COVID-19 vaccination amongst key populations, classify existing strategies/resources used by these PHUs and identify the barriers and enablers to vaccination that these strategies are designed to address. This behavioural science-informed scan is reported in a separate paper [58].

#### Steps 4 and 5 – Building relationships with each community, establishing the engagement framework and establishing partnerships with community members

**Fig. 2:**
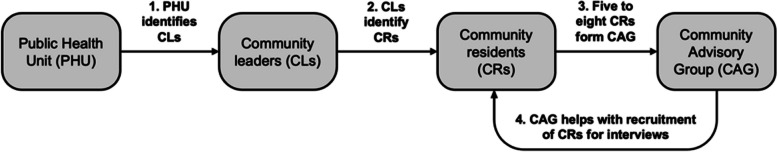
Approach to community engagement involving the Public Health Unit (PHU), community leaders, community residents and a Community Advisory Group (CAG)

The research team continued to build relationships with members from each community, following our planned approach to community engagement (see Fig. 2). This involved working with PHUs to identify community leaders or organizations who would then select members to form Community Advisory Groups (CAGs) for each city. Each CAG, comprising 5 to 10 members per PHU, collaborated with the team on key research activities, such as recruiting individuals for qualitative interviews. The aim of the CAGs was to guide the community engagement process, and support the investigator team in understanding unique neighbourhood health dynamics, and analyze the factors influencing healthcare behaviours, more specifically COVID-19 vaccination, in each community. When recruiting CAG members, we aimed for broad representation aligned with the characteristics of the public health priority populations, however there was no formal mechanism in place to ensure equitable representation according to sociodemographic factors.

During this process, we finalized the engagement framework, outlining the principles, values, and practices that guided the development of trust and the partnership between academic researchers and community members in this project. The community engagement framework in the OPTimise Platform was based on the Patient Engagement In Research (PEIR) Framework developed by Hamilton and colleagues [59] and on strategies identified by De Weger and colleagues (see Table 2) [60]. The PEIR Framework is an empirically based conceptual framework for effective PEIR founded on a patient perspective. The PEIR Framework includes eight key organizing themes: (1) procedural requirements, (2) convenience, (3) contributions, (4) team interaction, (5) research environment, (6) support, (7) feel valued, and (8) benefits. The guiding principles for community engagement in the OPTimise Platform were structured according to these themes. The main principles of the engagement framework were introduced during initial discussions with community leaders. Our framework emphasized mutual respect, shared decision-making, and measures to promote equitable partnerships, reduce power imbalances, and enhance the validity and relevance of research conducted.

**Table 2:** OPTimise platform community engagement framework, based on work by Hamilton and colleagues [59] and De Weger and colleagues [60]

Organizing themes	Guiding principles	Operationalization in OPTimise Platform
**1. Procedural requirements** Procedural details involved in managing the inclusion of community members in a research project to ensure their experiences are rewarding and productive	Ensuring sufficient and diverse representation	• We worked to identify community members who can represent the perspectives and interests of the priority group
	Clarifying roles	• From the onset, we discussed options for roles and tasks with community members to elicit their preferences and ensure they were comfortable with the level of engagement • These roles included: ◦ Assisting with development of our recruitment and interview materials ◦ Promoting the project and/or recruiting participants within their community ◦ Interpreting the results of the interviews ◦ Participating in the development of recommendations for Public Health Units
	Offering compensation	• Each community leader/resident was offered a set compensation amount for assisting with the project and compensation for additional contributions; we consulted with them to offer this compensation in a way that worked best for them (e.g., cheque, electronic or mailed gift cards)
	Using plain language	• We made sure the documents developed for community members were in plain language and, in some cases, translated in Arabic and French
**2. Convenience** Importance of choice and accessibility, including sufficient time to engage, and the flexibility to choose how and when to contribute	Ensuring accessibility	• We scheduled meetings at times convenient to community members and offered alternative ways to contribute (e.g., email, telephone calls) • We ensured there was sufficient time for contributing during meetings • We circulated project and meeting materials (e.g., slide decks, recruitment posters) through different communication platforms (e.g., email, WhatsApp) and created a shared online folder for all materials
	Ensuring flexibility	• We offered community members the opportunity to join the meeting through different means (e.g., joining Zoom by calling on telephone) • We clarified that we understand if not all meetings can be attended and offered individual meetings or telephone calls to cover missed material • We used different approaches to receive feedback (e.g., one on one conversations for feedback on interview guides) and encouraged the use of individuals’ preferred methods of communication (e.g., text, call, voice messages, email)
**3. Contributions** Roles and tasks assumed by community members	Providing constructive feedback	• We provided regular, constructive feedback on the roles and tasks assumed by community members; we explained how their feedback was shaping the project
**4. Team interaction** Importance of positive research team interaction	Identifying one person who can be contacted	• We identified one consistent “point” person on the research team whom community members could contact if they needed information or support
	Ensuring a reciprocal relationship and positive social interactions	• We engaged regularly with community members not only in a ‘research’ context, but also socially through informal conversations • We emphasized the importance of mutual respect and trust
**5. Research environment** Importance of having a positive and inclusive organizational/team culture that allows partners to feel comfortable and accepted as equal team members working together	Fostering a safe and trusting environment	• By clearly stating values of inclusiveness and respect from the onset, we fostered a safe and trusting environment enabling community members to provide input • The research team played a mediating role by encouraging honest feedback, actively listening and ensuring tensions could be openly discussed
	Acknowledging power imbalances	• We acknowledged and addressed community member experiences of power imbalances between citizens and health care professionals • We had discussions about what community members brought to the table (e.g., feedback, comments, expertise, background)
**6. Support** Financial support that covers engagement-related expenses and instructional support provided training to improve understanding of research language and processes	Reimbursing expenses related to project engagement	• In addition to compensating community members, we offered to reimburse any additional project engagement expenses (e.g., extra meetings)
	Providing skills/instructional support	• At the first meeting, we explained research language and procedures • We integrated training into our meetings based on the needs of the specific group (e.g., information about the role of research ethics boards, how this impacts recruitment and interviews) • We offered additional on-demand instructional support, including in the individual’s first language when possible
**7. Feel valued** Ensuring that community members feel equally important on the research team by demonstrating appropriate recognition and respect	Considering the community KUs’ motivations	• We explored the community members’ motivations for joining the project and considered how we can align the project with these motivations
	Acknowledging contributions	• We reviewed community member’s contributions and successes at each meeting and stressed the importance of their expertise • We demonstrated their impact on the project (e.g., “What we heard/What we did” – see Table 3)
	Creating quick and tangible wins	• We structured each meeting with community members to provide quick and tangible wins (e.g., collecting specific input about the interview guides that would help us move forward)
**8. Benefits** Importance of community members to derive benefits from their engagement	Highlighting the benefits of engagement	• We highlighted the benefits of engagement for community members, including gaining confidence, knowledge and skills to communicate their perspective in a research team and learning about COVID-19 and vaccination • We communicated how the research team had benefitted from their engagement (e.g., personal growth, better understanding of complex issues and challenges faced by their communities)
	Investing in citizens who are less often provided with opportunities to engage with researchers	• We provided engagement and learning opportunities to community members who felt they lacked the skills and confidence to engage • We offered to make them aware of further opportunities for engagement

*KU* knowledge user

#### Step 6 – Involving community members in the research process

From the onset, before partnerships were formalized and again during the first meeting of each CAG, we discussed options for the roles and tasks of community members to elicit their preferences and ensure that they were comfortable with their level of engagement. The tasks that members of CAGs could help us with included assisting with the development of our recruitment and interview materials, promoting the project and/or recruiting participants within their community, interpreting the analysis of the results of the qualitative interviews, reviewing and participating in the elaboration of recommendations for PHUs and supporting the dissemination of the results to their communities, the general public and other partners. Throughout the process, we produced short documents titled “What We Heard/What We Did” to summarize feedback received and explain what feedback we were able to incorporate and what we could not and why (see Table 3). This feedback mechanism proved useful for communicating scope of the project, the research process, and the requirements of the research ethics board. It also demonstrated to the CAGs that we were actively listening and valued their contributions.

**Table 3:** Example of a ‘What We Heard/What We Did’ document in the initial stage of the community engagement with the Ottawa CAG for recruitment posters

What we heard	What we did
**Conversations is a better term than « interviews»**	✓ From now on, we will use conversation! Thank you!
**The reading level is too high**	✓ We’ve decreased the reading level.
**There is too much information**	✓ We’re reduced the content by about 30%.
**Concerns with putting “vaccine” in the heading**	You made a good point about people being tired to speaking about vaccination, but research projects must follow very strict rules from a “Research Ethics Board” (a group of people, including citizens, who make sure that people who participate in research are protected). We must state the purpose of the interview very clearly up front.
**Concerns with putting 3rd dose/booster in the heading; people have lost track of what they have received so this could be confusing**	We must be clear that it is for 3^rd^ dose/booster. We can’t think of a simpler way of saying this.
**Add a QR code on the poster**	✓ We will do this
**We like the subheadings (What do I have to do?… We’d like to hear your thoughts about…)**	✓ Glad to hear this. We’ve kept them in the new version.
**Nice graphics, especially hands symbolizing collaboration**	✓ Thank you. We’ve kept the hands!
**The poster needs to be more colourful, with bigger logos & pictures, and less “institutional”**	✓ Great advice! We have made the poster more colourful.
**Make poster available in different languages (e.g., Arabic, French)**	✓ We will do this.

With support from the CAGs, we conducted theory-informed interviews guided by the Theoretical Domains Framework (TDF) and explored barriers and enablers to COVID-19 vaccination along 14 domains (e.g., environmental context and resources; social influences; emotion; behavioural regulation) [35, 61]. Individual interviews were preferred over focus groups; while focus group discussions can offer valuable insights into interpersonal and community dynamics, we were concerned about potential censoring of views in the presence of others and the influence of dominant voices in such settings particularly given the sensitive and politicized nature of COVID-19 vaccination. To mitigate the risk of bias and ensure a more comprehensive capture of community attitudes towards vaccination, we employed a purposive sampling strategy and stratified our sampling based on the number of COVID-19 doses received (unvaccinated, 1–2 doses, and 3 or more doses). Data saturation was assessed using Francis’ 10 + 3 rule for theory-informed interviews [62]. We conducted 22, 21, and 25 interviews in Ottawa, Peel and Toronto, respectively. These included 14 interviews with people who were not vaccinated, three with people who had the first dose, and 36 with people who had the second dose. Additionally, we interviewed 15 people who had 3 or more doses. Despite efforts to achieve more balanced samples, more women than men participated in interviews.

#### Step 7 – Feeding back and interpreting research findings

**Fig. 3:**
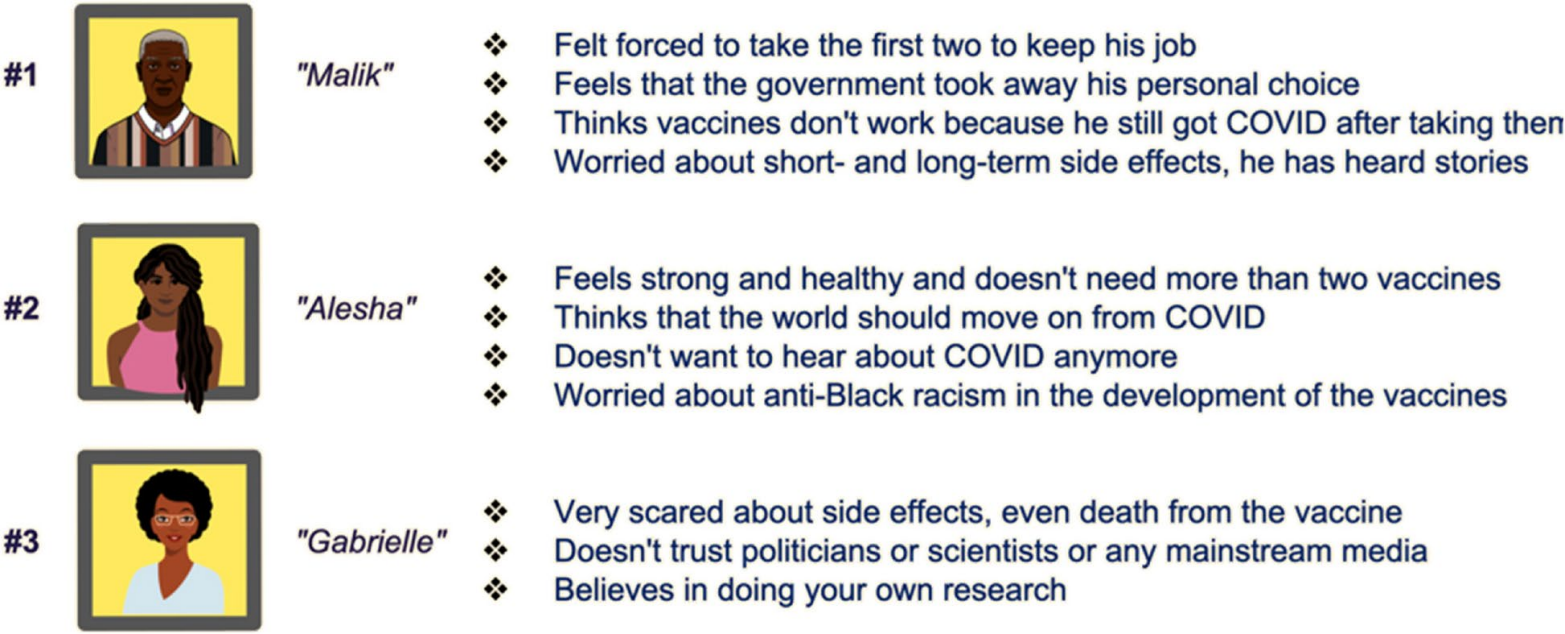
Personas used to guide discussions around strategies to promote the uptake of vaccination with the Toronto Community Advisory Group (CAG)

Once the qualitative interviews with members of each community were completed, additional meetings with CAGs were held to discuss and interpret findings. Individual interviews were triangulated with insights for the CAGs and existing literature. This triangulation helped in validating the insights gained from interviews and in constructing a more comprehensive picture of community dynamics and attitudes. Based on this data, we developed ‘personas’, who represented fictitious individuals in each community, as a way of presenting the themes and perspectives derived from the qualitative interviews (see Fig. 3). Three to four personas were created per community by the team members conducting data analysis, and these were then examined and refined by the community engagement co-leads. The CAGs recognized that the fictitious personas represented familiar perspectives of their neighbours and peers, which facilitated rich observations of what types of strategies could resonate with these personas.

When it was time to develop recommendations for PHUs based on the analysis of the results of qualitative interviews, we produced ‘What We Heard’ documents to present a summary of the feedback from each CAG from the previous meeting regarding each potential recommendation (see Table 4). These in-depth feedback sessions enabled members of each CAG to validate recommendations emerging from their community before they were shared with PHUs. At the end of the community engagement process, we held a final meeting with each CAG to reflect on our collective progress, areas of learning and mutual growth, and highlighting how their input directly influenced public health recommendations. Members of each CAG also received the findings of an independent evaluation of the engagement process to demonstrate the value of the study. Recommendations were then shared through policy briefs with key stakeholders from each PHU, and meetings were held to discuss recommendations. The PHUs received these briefs and recommendations favourably and spurred further follow-up discussions with PHUs on opportunities to action the recommendations, indicating that our community-engaged approach produced actionable insights tailored to their specific needs and contexts.

**Table 4:** Example of a ‘What We Heard’ document in the latter stage of the community engagement process with the Toronto CAG for recommendations to the PHU (details regarding each recommendation added to clarify recommendations)

Recommendation idea	What we heard
**Use windows of opportunity to start conversations** • Use March 11, 2023 (3rd anniversary of pandemic) to launch a new campaign about what we have learned about COVID and the vaccines	• Not sure if people will be receptive to a rebranding campaign • The word “anniversary” implies celebration: COVID is not something to celebrate
**Empower trusted sources** • Make sure people are aware of their important role in affecting the COVID-19 vaccination decisions that their patients, congregation, family, friends, and peers make	• It’s true that faith leaders have a lot of influence • Reach leaders through the higher-ups in the church system (the top dogs) Strategically choose churches for campaigns • Include Black health professionals and experts
**Roll with resistance** • Empower (through offers to support training) trusted sources to draw from the principles of motivational communication to keep the door open by “rolling with resistance” where the goals are to avoid defensiveness and encourage people see different perspectives	• This one is great • The non-judgmental piece is important • Respect people's right to make their own decisions
**Clarify key information** • Acknowledge that the messaging around COVID has been mixed/unclear, and clarify that it is less about how many doses you had and more about having a dose recently, so that your body is ready to fight COVID	• Good information, can accompany with visuals • Great use of language to explain things in a new way (immune system part) • Need to clarify what different variants mean
**Use stories alongside statistics** • Identify examples from within communities where people have changed their minds about the vaccine to amplify; stories from local community leaders, community ambassadors and relatable “regular people”	• Use videos, audio, people learn differently • In-person also very important e.g., wellness clinics • Could be playing on the screen at wellness clinics etc., people will watch while they’re waiting around

## Results

Our study yielded insightful findings on the complexities of aligning public health needs, community engagement and behavioural science research. We explored the nuances of working with diverse communities, and the critical role of trust-building in fostering effective partnerships. We outline here the lessons learnt in applying the methodology.

### Lessons learnt

#### One size doesn’t fit all: tailoring community engagement

Early in the engagement process, the research team recognized the necessity for tailored approaches for each community, considering the broader sociopolitical context, the polarization of views regarding COVID-19, the geographic distance of the research team to prioritised communities, and the distinct needs and cultural backgrounds of the communities. While the original strategy involved partnering with PHUs to identify community leaders or organizations, who would in turn help us select local residents to form CAGs for collaborative research in each city, this method proved unfeasible across all locations. Across all communities and at every step of the way, we had to consider the implications of working on a topic that became highly politicized with the potential for generating strong emotional reactions, influencing who ended up wanting to collaborate on this project. This necessitated tailoring the engagement process to each community, grounded in a fundamental respect for their diverse perspectives and needs. The study required adaptations to the envisioned approach, particularly in forming Community Advisory Groups (CAGs).

In Ottawa, we implemented the approach the research team had originally envisioned. The process began with meetings at the Ottawa PHU, where key partners helped identify individuals from diverse community organizations, such as Community Resource Centres (CRCs) and Community Health Centres (CHCs). These community leaders, holding roles like Community Development Facilitator, Health Promoter, COVID-19 Coordinator and Community Capacity Coordinator, identified residents to form a CAG. From the residents approached, a group of six individuals agreed to form the Ottawa CAG, establishing a strong local connection. Our research team’s citizen partner led Ottawa’s community engagement, utilizing skills as a second language teacher and a person who is experienced in bridging the gap between the public and researchers. This intermediary role between the researchers and the community leaders seemingly helped mitigate barriers to engaging in research (e.g., accessible language, understanding of research systems, tokenism) that people from equity-denied groups face.

In Toronto, it was necessary to adopt a different approach as the PHU did not directly connect us with community organizations related to the priority group. Despite extensive discussions with community leaders and several unsuccessful attempts at recruiting residents, we adapted our strategy due to historical mistrust and injustices, which were intensified by COVID-19. We cold-called relevant organizations and had discussions with a Black researcher in Toronto, which allowed us to identify eight community leaders from Black, African and Caribbean communities with connections to various organizations (e.g., Jamaican Canadian Association, Grenada Cultural Association, Community Health Centres) interested in forming a CAG. Most community leaders in the Toronto CAG did not live within the five neighbourhoods, but were members of African, Black, and Caribbean communities, and some worked in close proximity to the neighbourhoods.

In Peel, like in Ottawa, connections were established with community agencies and organizations through introductions from the PHU. However, this initiative intersected with the geopolitical upheaval following Russia's invasion of Ukraine in February 2022, leading to a pause in community engagement efforts. A breakthrough came in spring 2023 with introductions from the Peel PHU to community organizations, as was the case in Ottawa. This led to a successful Zoom presentation to about 45 organizations, which, along with PHU connections, enabled the formation of a Peel CAG with community residents. The CAG included eight community residents primarily of younger age from Ukrainian, Polish and Bosnian backgrounds. Several community partners were employed as staff at various community organizations in Peel, working specifically with the communities with whom we sought to connect, and several had left Ukraine because of the Russo-Ukrainian War. Although exact educational backgrounds were not explicitly detailed, many had some tertiary education. Several newcomer members viewed their participation as a means for professional advancement in Canada, with some offering indispensable language support for interviews.

Overall, the experiences in these regions underscore the importance of flexibility, responsiveness and cultural sensitivity in community engagement, particularly in diverse and dynamic sociopolitical contexts.

#### Involvement of the community advisory groups in the research process

Across sites, we used different methods of engaging community members in the research process. In Ottawa, our approach was shaped by the research readiness of our community members. Most were new Canadians and not familiar with research practices. To enable effective participation in the research process, each meeting included some type of training. Throughout our collaborative sessions, we discussed topics such as the importance of research ethics board requirements when co-creating recruitment documents or asking community members if they were interested in interviews. In later stages, our discussions were mostly focused on anecdotes and personal stories that reflected the community’s experiences and shaped our recommendations in a way that was close to the community.

In Toronto, where prioritized populations were African, Black, and Caribbean communities, our discussions reflected the community leaders’ expertise in their communities and were focused on intersectional issues and structures of power within society. They helped us understand the factors that influenced their community’s behaviours, what could potentially be done to repair relationships, and provided messaging that resonated with their communities. There were open and honest conversations about how unethical research and systemic racism continues to have profound repercussions on African, Black, and Caribbean communities’ relationships with the healthcare system, the government, and the scientific community. As health and social services professionals, community leaders shared how they interacted with people from those communities and what they heard about COVID-19 and vaccination. We were able to delve more deeply into behavioural science approaches as, in general, there were fewer barriers and differences related to language skills and overall health literacy.

The involvement of the Peel CAG was multifaceted, reflecting their cultural expertise, personal background and professional experience. To navigate the intricacies of the Eastern European community in Peel, members of the CAG offered valuable insights into the politics, history, and structures of power within various Eastern European countries. CAG meetings in Peel revealed substantial learnings about the cultural and political context, such as the influence of war in Ukraine and attitudes toward vaccination in other countries. Feedback from the CAG and community leaders emphasized a deeply rooted mistrust in government and its extensions, including research entities, due to historical political corruption in Eastern Europe. This necessitated building trust bridges, which the CAG aptly facilitated, highlighting the indispensable nature of their involvement in the research process. On a practical level, some CAG members actively participated in interviews by assisting with language interpretation, translating information ‘on-the-fly’, ensuring that the nuances were preserved.

#### Navigating diverse public health unit priorities

To effectively navigate the complexities of the OPTimise Platform, a wide range of questions emerged throughout the project (see Table 5). These guiding questions should be kept in mind from the onset of projects involving collaborations between PHUs and behavioural scientists. This process can generate tensions, therefore efficient, transparent communication is key to ensuring balance between the respective interests of each group. In the OPTimise Platform, this required not only a clear understanding of each party’s objectives but also a commitment to open dialogue and collaboration, ensuring that both the practical needs of the PHUs and the scientific goals of the researchers were adequately addressed and harmonized.

**Table 5:** Guiding questions when developing collaborations between behavioural scientists and public health units

1. What are the specific public health challenges faced by Public Health Units (PHUs), and how do these translate to their goals and needs?
2. How can applied behavioural science contribute to addressing these goals and needs (e.g., exploring barriers to vaccination, designing strategies to promote vaccination)?
3. What are the metrics of success for PHUs, and how can these be aligned with behavioural research findings?
4. How can the collaboration between PHUs and behavioural scientists be structured to ensure mutual benefit?
5. How can the partnership between PHUs and behavioural scientists remain flexible to adapt to new public health emergencies or changes in community health profiles?
6. What communication strategies can be employed to effectively convey the findings and benefits of behavioural science interventions to diverse stakeholders?
7. What are the best practices to build capacity within PHU staff in the principles and applications of behavioural science to enhance in-house expertise?

The prioritization discussions within the project revealed differing preferences among the PHUs for the types of support they needed. While one PHU was seeking assistance in reaching out to communities where establishing connections had proven challenging, the other two PHUs were more interested in working with communities where they had existing relationships but still faced low vaccine uptake rates. Furthermore, a significant aspect that came up during the prioritization process was defining what constituted a community in the context of the project. We adopted a broad definition of community, recognizing that communities may be arrayed along a spectrum of cohesiveness, with a different set of characteristics (e.g., common culture and traditions, canon of knowledge, and shared history; health-related common culture; legitimate political authority; representative group/individuals; mechanism for priority setting in health care; geographic localization; common economy/shared resources; communication network; self-identification as community) [63]. Consequently, our definition broadened to include not just geographic proximity but also shared identities, interests, cultural practices, and social connections. One PHU expressed a desire to focus on communities with low socio-economic status (SES), while the other PHUs preferred to focus on specific cultural population groups, acknowledging the unique challenges and opportunities in enhancing vaccine uptake within these communities. These distinct priorities shaped our approach to community engagement, tailoring it to the specific vaccination goals and the communities prioritized by each PHU.

#### Building trust for effective community engagement

The trust-building process in this study was crucial for effective community engagement and research partnerships, especially with communities impacted by discrimination, racism, and systemic inequities. This process was influenced by historical trauma, pre-existing mistrust towards researchers, healthcare professionals, and government, community governance, resources and challenges posed by COVID-19 and geographic distances. The team identified five key trust-building mechanisms based on their experience: (1) getting acquainted; (2) ensuring cultural and linguistic competence of the research team; (3) working out differences and resolving conflicts; (4) acknowledging the validity of mistrust and damaged relationships based on past experiences; and (5) addressing ethical considerations and ensuring reciprocity.

*Getting acquainted* was a crucial trust-building mechanism as it allowed for the establishment of mutual respect, understanding and shared goals between researchers and community members. This was achieved through the research team learning about the communities, engaging in active listening and demonstrating a genuine interest in the community's concerns and perspectives during meetings with community leaders.

*Ensuring the cultural and linguistic competence of the research team* was also fundamental since we engaged with people from diverse sociocultural backgrounds who spoke different languages and had varying levels of health and research literacy. Although the research team was prepared to do this, it was not until the PHUs were identified and the prioritised communities were chosen that we were able to address the cultural and linguistic competence of our team. Working with different groups from widely different sociocultural backgrounds required the team to be especially conscious of what they knew and also what they did not know, and to seek advice and to secure external support when required. We recruited team members fluent in different languages (e.g., Arabic, French, Ukrainian) to help support community engagement meetings and conduct the qualitative semi-structured interviews to identify barriers and enablers to vaccination uptake in the prioritised communities. Throughout the project, we consulted with community members to ensure culturally appropriate activities (e.g., acknowledging Black History Month, sensitivity in relation to the Russo-Ukrainian War).

*Working out differences and resolving conflicts* helped build and maintain trust among community members at different levels. This involved acknowledging and addressing power imbalances, communicating openly and honestly and using a collaborative problem-solving approach. For example, during the cold-calling process in Toronto we were confronted with questions from several intermediaries inquiring why, if we wanted to work with people from Black, African, and Caribbean communities, we did not have individuals from these communities represented on the research team. We carefully explained that the OPTimise Platform was designed to be agile and flexible in working with a wide range of communities, while recognising that indeed this was a limitation in the composition of our team and why we sought to partner. Thus, our plan was to engage members of the specific communities we would be working with before embarking on research activities to ensure local representation.

A central trust-building mechanism during our CAG meetings was *acknowledging the validity of mistrust and damaged relationships based on past experiences*. Acknowledging and addressing historical and systemic injustices was central to the community engagement process. Discussions with Toronto community leaders led us to understand that not all of them felt comfortable referring community residents to us to form a CAG due to the highly politicized and polarized nature of COVID-19, as well as historical mistrust in healthcare professionals and decision-makers. There were people sympathetic to anti-vaccine sentiments in the CAG; however, the research team and the CAG were able to engage in constructive discussions which respected the different perspectives. During our meetings with community leaders from Black, African and Caribbean communities, we discussed the historical and systemic injustices that have contributed to mistrust (e.g., the Tuskegee syphilis experiment) and worked to understand concerns and experiences. We learned a great deal about the impact of mandates (e.g., mandatory vaccination, travel restrictions) on communities and how it affected community relationships with health professionals.

A final trust-building mechanism, operationalized mainly when partnerships were established and the Ottawa, Peel and Toronto CAGs were formed, was *addressing ethical considerations and ensuring reciprocity*. Many ethical issues emerged that needed to be addressed when working with these equity-denied communities. These included achieving a true “community-driven” agenda, addressing insider–outsider tensions, racism, limitations of “participation,” as well as issues involving the sharing, ownership and use of findings [48, 64].

## Discussion

We developed and applied a dynamic community engagement approach involving close collaboration with PHUs, community leaders, and residents. This approach facilitated community-based behavioural science research with equity-denied groups to inform public health strategies to enhance vaccination uptake and curb the spread of COVID-19. After PHUs identified priority communities and vaccination targets, we collaborated closely with community members. This collaboration helped in the recruitment of their peers for theory-informed interviews and facilitated in-depth understanding of, and community-driven perspectives on, the factors influencing COVID-19 vaccination uptake, as well as interpretation of findings and co-design of timely recommendations tailored to each PHU in Ottawa, Peel and Toronto.

Our study shows why and how a ‘one size fits all’ approach to community engagement does not work when engaging with individuals from a wide range of sociocultural backgrounds, particularly around a highly politicized and polarized subject. The principle of ‘one size does not fit all’ starts at the study design and grant application stage; we must be nimble and prepared to adapt based on the community and their needs as they evolve throughout the project. While we aimed to adopt a rapid, agile approach to the current project given the pandemic context (including the use of a novel, rapid analysis approach for interviews with residents), it still took several months to recruit enough participants to achieve a desirable sample size in target populations, conduct the co-design process and produce recommendations to PHUs. It was imperative to tailor our approach to community engagement, carefully considering factors such as the specific partnerships researchers establish within each community, the cultural and linguistic nuances of the community (e.g., language skills and preferences, communication styles), the historical and social context surrounding the issue at hand, the accessibility of resources for community members, and the support systems and organizations within the community. Overall, this approach requires researchers to adopt a more flexible, empathetic, and community-centric perspective, moving away from a one-size-fits-all methodology and pre-established protocols. Although the urgency of a public health crisis can result in projects being conducted more quickly, it remains possible to build trusting relationships if meetings and feedback occur regularly and consistently while respecting the overall project timeline [41, 42, 55, 57]. Community members were understanding of the urgency surrounding the situation and agreed to shorter timelines when approached transparently. Trust can be established and maintained even in the face of time constraints by maintaining open lines of communication, and consistently involving community members in the decision-making process.

Approaches such as iKT and CBPR have enormous value in informing public health authorities, especially when working with historically excluded groups [42, 43, 45]. Building on the expertise of community members, gained through lived experience, is essential for designing public health strategies and policies that reflect the realities of those communities. Effective partnerships are founded on mutual respect for each other's expertise, with researchers and community members working collaboratively [41, 45]. Before embarking on any research involving equity-denied groups, researchers need to be aware of the barriers to participation based on past history, potential harms, pre-conceived notions about research and uneasiness with the roles of researchers within society (past and present) [64]. We have to change the widely held (and often justified) belief that the researcher will parachute in, take what they need and walk away from the community without concrete measures to give back to the community [64]. Research involving community engagement has immense value for addressing issues that are relevant to communities and for proposing solutions informed by the expertise of the end-users in these communities. “Nothing about us, without us” is at the heart of community engagement. The voices of diverse members of the community are especially important in non-disease specific topics like public health, where people are coming to the table to share lived experience as members of a community, rather than with a specific disease or condition. This project demonstrated that it is not simply a matter of translating the ‘tried and tested’ patient engagement strategies to public health community engagement. In the public health sphere, there remains a need for agile and tailored community engagement strategies, and the current study fills a key knowledge gap in linking behavioural science approaches to iKT and CBPR [65–67].

Presenting complex findings from behavioural science was pivotal in engaging CAGs in effective discussions in the OPTimise Platform. The use of ‘personas’ crafted as fictional individuals, each embodying the diverse themes and perspectives unearthed from the qualitative interviews, was deemed effective by CAG members. They not only recognized these personas as reflective of their community's diverse viewpoints but also found them relatable, akin to familiar neighbours and peers. This familiarity was crucial—it transformed our findings from abstract concepts into tangible, relatable narratives, fostering a deeper understanding among the CAGs. The personas served as a bridge, linking the theoretical aspects of our research with the practical, lived experiences of the community members. This, in turn, sparked rich discussions, enabling the CAGs to provide insightful observations and suggestions on potential strategies that could effectively resonate with the personas represented. Translating data into narratives that CAGs can connect with, we not only enhance their ability to engage with the findings but also empower them to contribute meaningfully to the discussion.

This study has some limitations that should be considered when interpreting the findings and considering the applicability of our approach to other contexts. First, our methodology was relatively resource-intensive, requiring significant time (around a year from the prioritization of key behaviours and populations by PHUs to the production of tailored recommendations) and effort from the research team and community partners. For many PHUs, this level of resource commitment may not be feasible, especially those with limited funding or staffing. However, we hope this approach also demonstrates the complementary–and at times, bridging–role that research teams can have in partnering with PHUs and communities, whereby research teams can provide complementary resources and expertise to PHUs. Furthermore, leveraging the methods and lessons from this process might improve efficiency in future applications. For future studies in low-resource settings, the use of existing and adaptable interview guides from this project could expedite data collection, as could partnering with research teams. Second, our recruitment strategy, which relied heavily on the existing networks of our community partners, may have introduced biases in the sample, study activities and outputs. While we made efforts to include diverse community members, there is a possibility that we missed individuals who are not connected to these networks or who face specific barriers related to trust, access to healthcare, and information. This could limit the generalizability of our findings to the broader community. Lastly, while we employed a variety of data sources to understand community-level perspectives including CAGs, we relied primarily on individual interviews and this can result in gaps in our understanding of the broader community context and concerns. Overall, while our approach provided valuable insights into community engagement and attitudes towards COVID-19 vaccination, readers should consider these limitations when adapting similar approaches to their own contexts.

## Conclusion

When community engagement results in a positive, rewarding experience, community members are more willing and capable of advocating for inclusion in research that concerns them, as well as communicating to researchers which factors contribute to effective participation in the research process. At various points throughout the study, community members shared positive feedback on their engagement experiences. Many community partners from all three geographical regions elected to be notified of future opportunities to collaborate with research teams. The findings from a formal third-party evaluation of the project’s citizen and knowledge user engagement will be published.

As citizen engagement in health research becomes more common, best practices need to reflect the diversity of communities and how the engagement approach must not be conceived as ‘one size fits all.’ The COVID-19 pandemic has put a spotlight on public health and the role of PHUs as key actors in keeping our communities safe. Moreover, increased public interest in science and public health since the outbreak of the COVID-19 pandemic, including both positive and negative perceptions, should be responded to by researchers with active engagement efforts. As researchers, we have an opportunity to highlight the value of community engagement and bring community members together to tackle complex public health issues such as climate change, environmental destruction and food insecurity. Combining community engagement and behavioural science approaches can result in public health policies and recommendations that are truly relevant and meaningful to diverse communities.

### Supplementary Information

**Supplementary Material 1.**

## Data Availability

All data generated or analysed during this study are included in this published article. This paper focuses on methodological reflections derived from community engagement in the OPTimise Platform. Given the nature of this work, which revolves around conceptual and methodological discourse, our emphasis was on the qualitative reflection and synthesis of community engagement practices. Results from the qualitative interviews conducted in the OPTimise Platform will be reported in a separate paper.

## Abbreviations

CAG: Community Advisory Group
CBPR: Community-Based Participatory Research
CHC: Community Health Centre
COVID-19: Coronavirus disease 2019
CRC: Community Resource Centre
iKT: Integrated Knowledge Translation
KU: Knowledge User
PEIR: Patient Engagement in Research Framework
PHU: Public Health Unit
