# Supplementary material for: One size doesn’t fit all: methodological reflections in conducting community-based behavioural science research to tailor COVID-19 vaccination initiatives for public health priority populations
Source: BMC Public Health. 2024 Mar 13;24:784. doi: 10.1186/s12889-024-18270-x (PMC10936009; doi:10.1186/s12889-024-18270-x)
Supplement: Supplementary file 1 — Supplementary Material 1. [file 12889_2024_18270_MOESM1_ESM.docx]

Supplementary File 1

**Criteria and process for identifying priority behaviours and groups**

**OPTimise Project**

*(finalized Jan 31, 2022)*

## Purpose

This project involves supporting Public Health Units (PHUs) by drawing from behavioural science to address current priority topics in their jurisdictions. This will involve PHUs prioritizing a key behaviour (e.g., vaccination, mask-wearing) and a key population group for whom they believe might benefit from new strategies to complement current PHU efforts to maximize population health and well-being within the context of the COVID-19 pandemic. **The first step in this process is** **identifying and prioritizing a behaviour and group in each city; this document describes a prioritization process and provides a matrix to facilitate this process**.

## Identification of criteria for prioritization

## We propose five base criteria from which each PHU will begin the selection of a priority behaviour and group for their city. The recommended criteria include:

## Uptake of behaviour in a population group is lower compared with other jurisdictions,

## PHU already engaged in activities with limited results on behaviour,

## Behaviour of interest is among an underserved or equity-deserving group,

## Behaviour or group aligns with PHUs trending concerns over the next 2-3 months, and

## Likely to have a positive impact.

Each PHU can add, remove, or refine criteria as appropriate/needed. However, the final criteria should include 3-5 criteria to guide prioritization.

## Select community health priorities

## The prioritization matrix below can be used by PHUs to list proposed options (i.e., the key behaviour and population group) and select criteria to identify PHU priorities. Each PHU should take the following steps to identify priorities:

## **Step 1**: identify a shortlist of local priority behaviours and groups in column 1

## **Step 2**: use criteria in other columns to indicate whether each potential behaviour and group meets the each criterion (indicate: yes, no, maybe)

## **Step 3**: use the final column to rank each option against the criteria (priority rank 1 will be the first behaviour/group focused upon for a given PHU).

Priority population groups may be defined by age, ethnic origin, gender, geographical region, language, etc. The following behaviours and population groups were identified at the time of grant submission, but each PHU is encouraged to consider whether the behaviour and group is still relevant at this time:

**Ottawa:** *COVID-19 vaccination in 18–39-year-olds from Black communities*

**Peel:** *Mask use in 20-29-year-olds*

**Toronto:**  *COVID-19 vaccination in 30–54 year-olds living in racialized communities in the northwest of Toronto*

|  | **Prioritization Criteria** | | | | | **Priority rank** |
| --- | --- | --- | --- | --- | --- | --- |
| **Potential behaviours & population groups** | **Uptake lower than expected** | **Existing connections** | **Underserved/equity-deserving group** | **Likely to have positive impact** | **Local focus for next 2-3 months** |  |
|  |  |  |  |  |  |  |
|  |  |  |  |  |  |  |
|  |  |  |  |  |  |  |
|  |  |  |  |  |  |  |
